# M. D’Arcet on the Employment of Gelatine and Fat of Bones as a Means of Ameliorating the Diet of the Poor

**Published:** 1842-05-07

**Authors:** 


*On the employment of the Gelatine and Fat of Bones as a means of ameliorating the diet of the poor.* By M. D'Arcet.—M. D'Arcet, from the examination of the annual reports of various hospitals, gives an apparently satisfactory answer to the seventh conclusion of the French Academy as to the nutritive power of gelatine. He shows, that the directors of the different hospitals at Lille, Metz, Lyons, Strasbourg, different parts of Russia, and the whole of Holland, have found experimentally, that the health of their inmates has improved from the date of their fitting up an apparatus for extracting the gelatine and fat from bones. With regard to one of the hospitals at Lyons, he mentions the very striking fact, that the mortality appeared to be reduced by this means alone from 90 to 72, at which it has averaged ever since the apparatus for the preparation of the gelatine soup was fitted up. Many other interesting facts are mentioned, showing the advantage of hospitals being provided with an apparatus for the preparation of gelatine from bones, for it appears that, whenever this gelatinous soup has been added to the diet of the patient, the sickness, and consequent expense for medicines has been greatly lessened, the mortality has been considerably diminished, and the general health of the inmates greatly improved.—*Ibid.*, from *Annales D'Hygiene Publique.* April, 1841.

